# Why are the fastest runners of intermediate size? Contrasting scaling of mechanical demands and muscle supply of work and power

**DOI:** 10.1098/rsbl.2020.0579

**Published:** 2020-10-07

**Authors:** J. R. Usherwood, N. W. Gladman

**Affiliations:** Structure and Motion Lab., The Royal Veterinary College, North Mymms, Hatfield, Herts, AL9 7TA, UK

**Keywords:** speed, gait, running, muscle, work, power

## Abstract

The fastest land animals are of intermediate size. Cheetah, antelope, greyhounds and racehorses have been measured running much faster than reported for elephants or elephant shrews. Can this be attributed to scaling of physical demands and explicit physiological constraints to supply? Here, we describe the scaling of mechanical work demand each stride, and the mechanical power demand each stance. Unlike muscle stress, strain and strain rate, these mechanical demands cannot be circumvented by changing the muscle gearing with minor adaptations in bone geometry or trivial adjustments to limb posture. Constraints to the capacity of muscle to supply work and power impose fundamental limitations to maximum speed. Given an upper limit to muscle work capacity each contraction, maximum speeds in big animals are constrained by the mechanical work demand each step. With an upper limit to instantaneous muscle power production, maximal speeds in small animals are limited by the high power demands during brief stance periods. The high maximum speed of the cheetah may therefore be attributed as much to its size as to its other anatomical and physiological adaptations.

## 1. Introduction

Greyhounds, racehorses and especially cheetahs are, in absolute terms, fast for terrestrial animals. Much smaller and larger animals have not been recorded achieving such high speeds—though reliable measurements of maximal speeds are notoriously difficult to obtain in animals that have not been bred and trained for racing. But, if we accept that intermediate-sized animals are indeed the fastest, how might this be explained? A range of evolutionary, ecological and sampling considerations may be pertinent. For instance, there may be little selective pressure for the largest animals to run fast in order to evade predators; there are very few species of very big animals from which to find a speed specialist. However, it may instead be that high absolute running speeds in very large or very small animals are impossible because of fundamental mechanical and physiological issues.

Historically, principles of scaling were applied to structural issues—the detail depending on mechanical features assumed to be of importance—predicting increasing speed at larger sizes [1,2]. Dynamic and geometric similarity [3] and energetic arguments [4] also point to increasing maximal speed capacity *V* with mass *m*, at *V* ∝ *m*^1/6^, though with little in the way of explicit mechanism*.* Some other mechanical constraint, such as strength [5–7] or disproportionate rate of fatigue [8], may then be invoked to account for the reduction in maximal speed at the largest sizes. However, it is unclear which constraints should be most influential, and in many cases why the scaling constraints imposed by these factors could not be circumvented with minor, otherwise inconsequential, deviations from some aspects of similarity. Further, it is not generally clear why there should ever be a transition between ‘too small’ and ‘too big' regimes: why does whatever balance between strength, deflection, inertia, fatigue etc. not end up predicting a constant scaling relationship? Some account has to be made for a change in scaling relationship with size, presumably relating to a transition in mechanical or physiological regimes.

Here, we approach the question by addressing the scaling implications of demand—specifically, of mechanical work each stride and of mechanical power during stance—and supply of muscle work, muscle power and physiological supply for muscle activation. We follow recent work accounting for scaling phenomena [9] ranging from posture [10] to the kinetics of young children [11] to the flapping and bounding flight of birds [12] by assuming that muscle is limited in its capacity to supply work and power.

## 2. Assumptions and model development

### (a) Muscle supply

We do *not* assume rigid geometric similarity, though we do assume locomotor muscle mass constitutes a constant proportion of body mass. Where alternative approaches might focus on the scaling of muscle stresses, strains and strain rates given geometric scaling assumptions, we assume that appropriate adjustments to internal/external moment arms and subsequent muscle ‘gear ratio' or ‘effective mechanical advantage' can be achieved with trivial adaptation of bone geometry and/or posture. However, certain muscle properties are constrained by fundamental biochemical processes. Here, we treat the internal workings of the limb as a suitably tuned black box and view the following properties to be ‘uncheatable’ with gearing, and mechanistically revealing: per muscle mass, we assume

(a) a constrained maximum work per contraction,
(b) a constrained power during the contraction,

and also consider the implications if there is

(c) a limiting physiological capacity to power muscle activation.

These assumptions are clearly incorrect in detail: the muscles of small, fast animals may be relatively fast and powerful. If peak muscle stress is considered scale-invariant [13–17], then the scaling of peak strain rate reported for fast muscles [13,16,18] indicates muscle power to scale with body mass as $\propto \;m^{-0.07\;\mathrm{to}\;-0.1}$, though the generality of this relationship might be questioned given recent measurements showing that single muscle fibres from big cats and rabbits have similar powers [19,20]. In any case, we assume here the extent of scaling of properties of the muscles recruited in highest speed locomotion of the fastest animal of each size is sufficiently dwarfed by the scaling of mechanical work and power demand across the size range of legged mammals to be negligible.

### (b) Stride frequency

We assume geometric similarity (isometry) applies to gross external form such that leg length *L* ∝ *m*^1/3^, and that stride frequency at maximal speed broadly follows dynamic similarity [21] such that

2.1 $$f\propto \sqrt{\frac{g}{L}}\propto L^{-1/2}\propto m^{-1/6},$$

and the inverse of this is stride period *T*_stride_, with

2.2 $$T_{\mathrm{stride}}\propto \sqrt{L}.$$

While reliable data for gait kinetics at close to maximal speeds are sparse, this scaling of stride frequency is supported by empirical observations of dogs and horses at high racing speeds. Greyhounds of approximately 35 kg use stride frequencies of 3.5–3.6 Hz (at 18–19 m s^−1^) [22,23]; Thoroughbred racehorses of mean mass 476 kg use stride frequencies of 2.3 Hz (at 17 m s^−1^) [24]. Dynamic and geometric similarity would result in constant stride frequency once normalized appropriately using $\hat{\;f_{m}}$:

2.3 $$\hat{\;f_{m}}=f\sqrt{\frac{m^{1/3}}{g}},$$

which provides values of 2.02–2.07 for greyhounds and 2.05 for the racehorses. Measurements of high-speed gaits at the more extreme ends of the mammalian size scale further indicate the lack of a strong scaling in $\hat{\;f_{m}}$. Wild brown rats, *Rattus norvegicus*, filmed in the field (120 Hz frame rate, Nikon Z6; 15 sequences, at least two large adults), of mass estimated at 400–500 g, had stride frequencies up to 6.7 Hz; $\hat{\;f_{m}}=1.83-1.90$. Asian elephants, *Elephas maximus*, (2790 kg) at high speed had stride frequencies of 1.5 Hz [25]; While frequency clearly varies with speed for a given animal, $\hat{\;f_{m}}$ near to maximal running speed appears broadly constant. We acknowledge this assumption will again be untrue in detail, but the consequences of deviation may then be considered within the context of the models presented here.

### (c) Mechanical power demands

**Figure 1.**
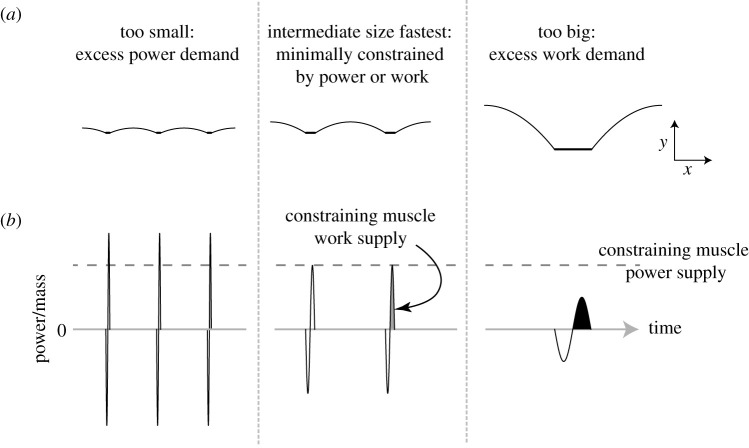
Cartoon generic centre of mass running paths (*a*) and mechanical power profiles (*b*) for small, intermediate and large animals at constant, high speed. Note that the geometric and scaling principles are not dependent on leg number; it is convenient here to display the geometry for a single-legged hopper. The positive mechanical work demand per distance travelled is the same at each size. At a given absolute speed, smaller animals have a higher step frequency, lower work each stance, but also much briefer stances resulting in higher peak power demands. The muscles of small animals cannot supply the power demanded at the highest running speeds. Larger animals have a lower step frequency, so higher work demands each stance. The muscles of very big animals cannot supply the mechanical work demanded at the highest running speeds. Between too-small and too-large, the fastest terrestrial animals occupy a size range that is minimally constrained by either work each contraction or power during stance.

We exploit the empirical relationship observed for a range of animals [26] for the rate of mechanical work of the centre of mass *P*_mech,CoM_ (from forceplate observations) as a function of size and speed. We do note that the empirical range of animals and of speeds measured is far from complete; however, we assume that the general observation holds for maximal speeds and across all mammal sizes:

2.4 $$\frac{P_{\mathrm{mech},\mathrm{CoM}}}{m}\propto V;$$

the mechanical power demand (per body mass) of locomotion varies in proportion to speed but ‘does not change in any regular way with body size' [26]. We do not propose a complete account for this observation, but note that:

(a) it is consistent with dynamic and geometric similarity for animals of different sizes [21]: work demand each step scales in proportion to leg length, as does step length, so work/distance is constant, as is power/velocity;
(b) at a given speed, larger animals take fewer strides per distance but, with lower stride frequencies, contact the ground with steeper trajectories (figure 1). Conversely, smaller animals at a given speed make more ground contacts per distance, and so have more occasions of mechanical loss and demand, but each contact—because of their higher step frequencies—has a shallower trajectory, reducing the work demand each contact.

## 3. The challenge with greater size: increasing mechanical work demand, constrained muscle work supply, per contraction

The work demanded each stride—or for each muscle contraction—is the product of the mean mechanical power demand (equation (2.4)) and the duration of the stride (equation (2.2)). Assuming a constraining, constant maximal muscle work supply per mass available each contraction at maximum speed *V*_lim,W_ (see also [27]):

3.1 $$\frac{P_{\mathrm{mech},\mathrm{CoM}}}{m}T_{\mathrm{stride}}\propto V_{\lim}\sqrt{L}=\text{constant, }$$

which predicts a work demand/supply constraint relationship for maximal running speed:

3.2 $$V_{\lim,W}\propto L^{-1/2}\propto m^{-1/6}.$$

## 4. One power challenge with small size: increased stance power demand, constrained instantaneous muscle power supply

Stance duration is constrained by geometry—the body cannot travel more than double the leg length each stance. Assuming something less than the splits is performed each stance (and this proportion does not scale with size):

4.1 $$T_{\mathrm{stance}}\propto \frac{L}{V}.$$

If the mechanical demand can only be supplied when the leg is loaded, with the foot on the ground, the stance power demand *P*_stance,D_ depends on both the mechanical work demand ((3.1), the product of (2.2) and (2.4)) and the stance duration *T*_stance_ (4.1) In this case, assuming a constraining, constant maximal muscle power supply available, matching the mechanical power demand during stance to the muscle power supply provides a second constraint relationship for maximal running speed *V*_lim,P_:

4.2 $$P_{\mathrm{stance},D}\propto \frac{VT_{\mathrm{stride}}}{T_{\mathrm{stance}}}\propto V\sqrt{L}\frac{V}{L}=\text{constant}$$

and

4.3 $$V_{\lim,P}\propto L^{1/4}\propto m^{1/12}.$$

## 5. A second power challenge with small size: activation power?

The metabolic work associated with activating and deactivating muscle can be a large proportion of the total demand, particularly for brief contractions (see [28]). We speculate that the supply meeting the rate of this demand *P*_act,D_ is fundamentally limited. If the activation demanded each contraction is sufficient to provide the stance power (4.2) due to contractions at a rate of stride frequency (2.1),

5.1 $$P_{\mathrm{act},D}\propto P_{\mathrm{stance},D}f\propto V^{2}\frac{1}{\sqrt{L}T_{\mathrm{stride}}}\propto \frac{V^{2}}{L},$$

resulting in a third constraint relationship for maximum running speed *V*_lim.act_, this time due to activation power:

5.2 $$V_{\lim,\mathrm{act}}\propto \sqrt{L}\propto m^{1/6}.$$

## 6. Parameterizing the model with the cheetah

The proportionalities (3.2), (4.3) and (5.2) can be turned into predictive equations provided three constants, one relating to the work rate constraint *k*_W_, the second to the stance power constraint *k*_P_ and the third to the activation power constraint *k*_act_:

6.1 $$\;\;\;V_{\lim,W}=k_{W}m^{-1/6},$$

6.2 $$\;\;\;\;V_{\lim,P}=k_{P}m^{1/12}$$

6.3 $$\text{and}\;V_{\lim,\mathrm{Act}}=k_{\mathrm{Act}}m^{1/6}.$$

**Figure 2.**
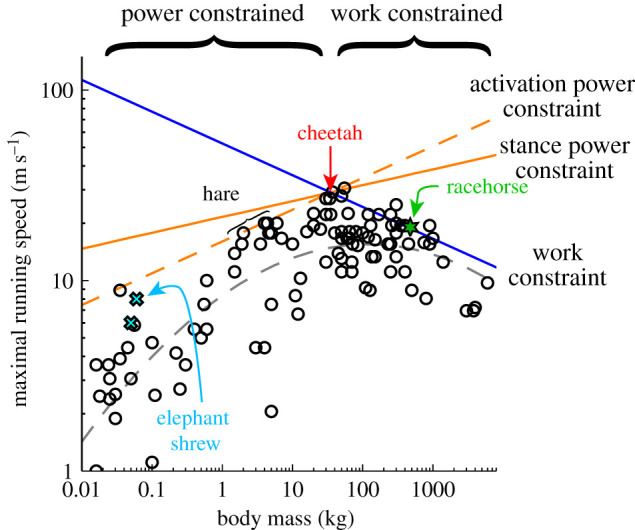
Model predictions due to constraints in muscle work (blue line), muscle power during stance (orange line) and physiological power supplying activation (dashed orange line), with reported maximal running speeds (circles) and regression fit (black dashed curve) [30] for animals of a range of sizes. The cyan crosses denote elephant shrews [31]; the green star racehorse (maximum 19.05 m/s, [32]). Model lines are parameterized using the relatively reliable observation of a cheetah [28]: *V* = 29 m/s; *m* = 35 kg, assuming this to represent the top animal speed, at the minimally constrained intermediate size.

These constants can be derived if velocity and mass are known at the intersection of the constraint lines. We assume the cheetah to be at this minimally constrained mass and are fortunate that a reasonably reliable measurement exists for something that must be approaching top speed [29]: 29 m s^−1^ for a female estimated to be 35 kg. From this datum, *k*_W_ = 52.45 m s^−1^ kg^1/6^, *K*_P_ = 21.6 m s^−1^ kg^−1/12^ and *k*_Act_ = 16.03 m s^−1^ kg^−1/6^. Predicted maximum velocities due to the three constraints are presented along with Garland's [30] maximum speed survey and a couple of more recent, perhaps more reliable, data points for elephant shrews [31] and Thoroughbred horses during racing [32] (figure 2; electronic supplementary material).

## 7. Discussion

Given the uncertainty surrounding many of the empirical speed measurements [30,31], and the sweeping nature of the assumptions to the work and power demand and supply models, it would be inappropriate at this stage to put too much emphasis on the detail of the model fit. Indeed, it is even unclear what should be viewed as a good fit: if only maximum possible speed at each size is of interest, perhaps something matching the upper convex hull of speed values? This is not the approach taken by Garland [30], who emphasizes the uncertain accuracy of measured values. But the polynomial fit is also difficult to justify, as the species selected—while subjectively fast—are otherwise arbitrary.

The scaling relationships for maximum speed limits presented here provide intuitive and mechanistic accounts for the deterioration of maximal absolute speed at very large and small scales (figure 1). Very big animals cannot supply the muscle work each stride required for very high speeds. Small animals would only achieve absolutely high running speeds with disproportionately low stance durations and are therefore prevented from very high speeds owing to the power demands during stance. While both proposed power constraints predict a reduction in maximal running speed at smaller sizes, the activation power constraint provides a closer fit to empirical observations. However, a fundamental constraint to muscle power is simple to justify, whereas a limit to supplying physiological power for activation is more speculative. At this stage, it may be best to conclude that the relatively high instantaneous power demands due to brief stances of small animals do provide a mechanistic account for reduced maximum running speed in small animals, but the details of constraining physiology are yet to be fully elucidated.

This reasoning developed here contrasts with the prevailing explanations, which develop a range of similarity-based accounts generally resulting in predictions of increased maximal speed with size, and then invoke structural constraints to account for the drop in maximal speeds at very large sizes. The mechanical demand versus muscle supply account developed here, albeit resulting in contrasting constraints, has the advantage of providing a parsimonious explanation within a single mechanistic framework; the cause of the transition in scaling regimes is explicit.

While measured muscle powers of cheetah, lion and leopard are high, at least in comparison with their respective prey species [19], they are not notably higher than those for wild rabbits [20]. Why, then, can the big cats certainly outpace the rabbit despite similar power supply capacity from the muscle? We suggest this is because of the very low stance durations resulting in high stance power, and high stride frequency resulting in high muscle activation power demands that would be required from a 20 m s^−1^ rabbit. The high top running speed of the cheetah can therefore be attributed as much to its intermediate size as to its other anatomical and physiological adaptations.

## Supplementary Material

Data and model used in Figure 2

## Data accessibility

Data presented in this paper are as stated in the text for stride timings: Usherwood & Wilson [22], Hudson *et al*. [23], Witte *et al*. [24], Hutchinson *et al*. [25], Rats: original data as stated. The speed data of figure 2 come from Garland [30], Lovegrove & Mowoe [31] and Spence *et al*. [32], The model and speed data are given in the electronic supplementary material.

## References

[1] Gunther B 1975 Dimensional analysis and theory of biological similarity. Physiol. Rev. 55, 659–699. (10.1152/physrev.1975.55.4.659) 1103169

[2] McMahon TA 1975 Using body size to understand the structural design of animals: quadrupedal locomotion. J. Appl. Physiol. 39, 619–627. (10.1152/jappl.1975.39.4.619) 1194153

[3] Schmidt-Nielsen K, Knut SN 1984 Scaling: why is animal size so important? Cambridge, UK: Cambridge University Press.

[4] Bejan A, Marden JH 2006 Unifying constructal theory for scale effects in running, swimming and flying. J. Exp. Biol. 209, 238–248. (10.1242/jeb.01974) 16391346

[5] Currey JD 1977 Mechanical properties of mother of pearl in tension. Proc. R. Soc. Lond. B 196, 443–463. (10.1098/rspb.1977.0050)

[6] Iriarte-Diaz J 2002 Differential scaling of locomotor performance in small and large terrestrial mammals. J. Exp. Biol. 205, 2897–2908. 12177154 10.1242/jeb.205.18.2897

[7] Fuentes MA 2016 Theoretical considerations on maximum running speeds for large and small animals. J. Theor. Biol. 390, 127–135. (10.1016/j.jtbi.2015.10.039) 26646766

[8] Hirt MR, Jetz W, Rall BC, Brose U 2017 A general scaling law reveals why the largest animals are not the fastest. Nat. Ecol. Evol. 1, 1116–1122. (10.1038/s41559-017-0241-4) 29046579

[9] Usherwood JR 2016 The muscle-mechanical compromise framework: implications for the scaling of gait and posture. J. Hum. Kinetics 52, 107–114. (10.1515/hukin-2015-0198) PMC5260522 28149398

[10] Usherwood JR 2013 Constraints on muscle performance provide a novel explanation for the scaling of posture in terrestrial animals. Biol. Lett. 9, 20130414 (10.1098/rsbl.2013.0414) 23825086 PMC3730652

[11] Usherwood JR, Hubel TY, Smith BJH, Davies ZTS, Sobota G 2018 The scaling or ontogeny of human gait kinetics and walk-run transition: the implications of work vs. peak power minimization. J. Biomech. 81, 12–21. (10.1016/j.jbiomech.2018.09.004) 30316545 PMC6224478

[12] Usherwood JR 2016 Physiological, aerodynamic and geometric constraints of flapping account for bird gaits, and bounding and flap-gliding flight strategies. J. Theor. Biol. 408, 42–52. (10.1016/j.jtbi.2016.07.003) 27418386 PMC5042028

[13] Marsh RL 1988 Ontogenesis of contractile properties of skeletal-muscle and sprint performance in the lizard *Dipsosaurus dorsalis*. J. Exp. Biol. 137, 119–139. 3209964 10.1242/jeb.137.1.119

[14] Seow CY, Ford LE 1991 Shortening velocity and power output of skinned muscle-fibers from mammals having a 25,000-fold range of body-mass. J. Gen. Physiol. 97, 541–560. (10.1085/jgp.97.3.541) 2037839 PMC2216485

[15] Marx JO, Olsson MC, Larsson L 2006 Scaling of skeletal muscle shortening velocity in mammals representing a 100,000-fold difference in body size. Pflugers Arch. Eur. J. Physiol. 452, 222–230. (10.1007/s00424-005-0017-6) 16333661

[16] Rome LC, Sosnicki AA, Goble DO 1990 Maximum velocity of shortening of three fibre types from horse soleus muscle: implications for scaling with body size. J. Physiol. Lond. 431, 173–185. (10.1113/jphysiol.1990.sp018325) 2100306 PMC1181769

[17] Rome LC 1992 Scaling of muscle-fibers and locomotion. J. Exp. Biol. 168, 243–252. 1640186 10.1242/jeb.168.1.243

[18] James RS, Cole NJ, Davies MLF, Johnston IA 1998 Scaling of intrinsic contractile properties and myofibrillar protein composition of fast muscle in the fish *Myoxocephalus scorpius* L. J. Exp. Biol. 201, 901–912. 9487095 10.1242/jeb.201.7.901

[19] Wilson AM et al. 2018 Biomechanics of predator–prey arms race in lion, zebra, cheetah and impala. Nature 554, 183–188. (10.1038/nature25479) 29364874

[20] Curtin NA, Diack RA, West TG, Wilson AM, Woledge RC 2015 Skinned fibres produce the same power and force as intact fibre bundles from muscle of wild rabbits. J. Exp. Biol. 218, 2856–2863. (10.1242/jeb.121897) 26206354

[21] Alexander RM, Jayes AS 1983 A dynamic similarity hypothesis for the gaits of quadrupedal mammals. J. Zool. 201, 135–152. (10.1111/j.1469-7998.1983.tb04266.x)

[22] Usherwood JR, Wilson AM 2005 Biomechanics: no force limit on greyhound sprint speed Nature 438, 753–754. (10.1038/438753a) 16341003

[23] Hudson PE, Corr SA, Wilson AM 2012 High speed galloping in the cheetah (*Acinonyx jubatus*) and the racing greyhound (*Canis familiaris*): spatio-temporal and kinetic characteristics J. Exp. Biol. 215, 2425–2434. (10.1242/jeb.066720) 22723482

[24] Witte TH, Hirst CV, Wilson AM 2006 Effect of speed on stride parameters in racehorses at gallop in field conditions J. Exp. Biol. 209, 4389–4397. (10.1242/jeb.02518) 17050854

[25] Hutchinson JR, Schwerda D, Famini DJ, Dale RHI, Fischer MS, Kram R 2006 The locomotor kinematics of Asian and African elephants: changes with speed and size J. Exp. Biol. 209, 3812–3827. (10.1242/jeb.02443) 16985198

[26] Heglund NC, Cavagna GA, Taylor CR 1982 Energetics and mechanics of terrestrial locomotion. 3. Energy changes of the center of mass as a function of speed and body size in birds and mammals. J. Exp. Biol. 97, 41–56. 7086349 10.1242/jeb.97.1.41

[27] Hill AV 1950 The dimensions of animals and their muscular dynamics. Sci. Prog. 38, 209–230.

[28] Barclay CJ 2015 Energetics of contraction. Comp. Physiol. 5, 961–995. (10.1002/cphy.c140038) 25880520

[29] Sharp NCC 1997 Timed running speed of a cheetah (*Acinonyx jubatus*). J. Zool. 241, 493–494. (10.1111/j.1469-7998.1997.tb04840.x)

[30] Garland T 1983 The relation between maximal running speed and body-mass in terrestrial mammals J. Zool. 199, 157–170. (10.1111/j.1469-7998.1983.tb02087.x)

[31] Lovegrove BG, Mowoe MO 2014 The evolution of micro-cursoriality in mammals J. Exp. Biol. 217, 1316–1325. (10.1242/jeb.095737) 24436375

[32] Spence AJ, Thurman AS, Maher MJ, Wilson AM 2012 Speed, pacing strategy and aerodynamic drafting in Thoroughbred horse racing Biol. Lett. 8, 678–681. (10.1098/rsbl.2011.1120) 22399784 PMC3391435

